# Clinical validity of the Italian adaptation of the Uniform Data Set Neuropsychological Test Battery (I-UDSNB) in Mild Cognitive Impairment and Alzheimer’s Disease

**DOI:** 10.1186/s13195-024-01465-0

**Published:** 2024-05-04

**Authors:** Francesca Conca, Valentina Esposito, Eleonora Catricalà, Rosa Manenti, Federica L’Abbate, Davide Quaranta, Guido Maria Giuffrè, Federica Rossetto, Federica Solca, Beatrice Orso, Emanuela Inguscio, Valeria Crepaldi, Maddalena De Matteis, Emanuela Rotondo, Marina Manera, Giulia Caruso, Valentina Catania, Elisa Canu, Francesco Rundo, Matteo Cotta Ramusino, Massimo Filippi, Cira Fundarò, Federica Piras, Andrea Arighi, Pietro Tiraboschi, Michelangelo Stanzani Maserati, Matteo Pardini, Barbara Poletti, Vincenzo Silani, Camillo Marra, Sonia Di Tella, Maria Cotelli, Raffaele Lodi, Fabrizio Tagliavini, Stefano Francesco Cappa

**Affiliations:** 1 ICoN Cognitive Neuroscience Center, Institute for Advanced Studies, IUSS, Pavia, Italy; 2 IRCCS Mondino Foundation, Pavia, Italy; 3 Neuropsychology Unit, IRCCS Istituto Centro San Giovanni di Dio Fatebenefratelli, Brescia, Italy; 4 Neurology Unit, Fondazione Policlinico Universitario “A. Gemelli” IRCCS, Rome, Italy; 5 Department of Psychology, Catholic University of the Sacred Heart, Milan, Italy; 6 IRCCS Fondazione Don Carlo Gnocchi, ONLUS, Milan, Italy; 7 Department of Neurology and Laboratory of Neuroscience, IRCCS Istituto Auxologico Italiano, Milan, Italy; 8 Department of Neuroscience, Rehabilitation, Ophthalmology, Genetics, Maternal and Child Health (DINOGMI), University of Genoa, Genoa, Italy; 9 Fondazione IRCCS Istituto Neurologico Carlo Besta, Milan, Italy; 10 IRCCS Istituto delle Scienze Neurologiche di Bologna, Bologna, Italy; 11 Neurodegenerative Diseases Unit, Fondazione IRCCS Ca’ Granda Ospedale Maggiore Policlinico, Milan, Italy; 12 Istituti Clinici Scientifici Maugeri IRCCS, Psychology Unit Pavia-Montescano, Pavia Institute, Pavia, Italy; 13 Neuropsychiatric Laboratory, Clinical Neuroscience and Neurorehabilitation Department, IRCCS Fondazione Santa Lucia, Rome, Italy; 14 Oasi Research Institute-IRCCS, Troina, Italy; 15 Neuroimaging Research Unit, Division of Neuroscience, IRCCS San Raffaele Scientific Institute, Milan, Italy; 16 Neurology Unit, Neurophysiology Service, Neurorehabilitation Unit, IRCCS San Raffaele Scientific Institute, Milan, Italy; 17 Vita-Salute San Raffaele University, Milan, Italy; 18 Istituti Clinici Scientifici Maugeri IRCCS, Neurophysiopatology Unit Pavia-Montescano, Pavia Institute, Pavia, Italy; 19 IRCCS Ospedale Policlinico S. Martino, Genoa, Italy; 20 Department of Oncology and Hemato-Oncology, Università degli Studi di Milano, Milan, Italy; 21 “Dino Ferrari” Center, Department of Pathophysiology and Transplantation, Università degli Studi di Milano, Milan, Italy

**Keywords:** Neuropsychological tests, UDS, Alzheimer’s Disease, Mild Cognitive Impairment, Cognition

## Abstract

**Background:**

The identification and staging of Alzheimer’s Disease (AD) represent a challenge, especially in the prodromal stage of Mild Cognitive Impairment (MCI), when cognitive changes can be subtle. Worldwide efforts were dedicated to select and harmonize available neuropsychological instruments. In Italy, the Italian Network of Neuroscience and Neuro-Rehabilitation has promoted the adaptation of the Uniform Data Set Neuropsychological Test Battery (I-UDSNB), collecting normative data from 433 healthy controls (HC).

Here, we aimed to explore the ability of I-UDSNB to differentiate between a) MCI and HC, b) AD and HC, c) MCI and AD.

**Methods:**

One hundred thirty-seven patients (65 MCI, 72 AD) diagnosed after clinical-neuropsychological assessment, and 137 HC were included. We compared the I-UDSNB scores between a) MCI and HC, b) AD and HC, c) MCI and AD, with t-tests. To identify the test(s) most capable of differentiating between groups, significant scores were entered in binary logistic and in stepwise regressions, and then in Receiver Operating Characteristic curve analyses.

**Results:**

Two episodic memory tests (Craft Story and Five Words test) differentiated MCI from HC subjects; Five Words test, Semantic Fluency (vegetables), and TMT-part B differentiated AD from, respectively, HC and MCI.

**Conclusions:**

Our findings indicate that the I-UDSNB is a suitable tool for the harmonized and concise assessment of patients with cognitive decline, showing high sensitivity and specificity for the diagnosis of MCI and AD.

**Supplementary Information:**

The online version contains supplementary material available at 10.1186/s13195-024-01465-0.

## Background

The identification and staging of individuals with Alzheimer’s Disease (AD) still represent a major challenge for clinicians, especially in the prodromal stage of Mild Cognitive Impairment (MCI), when cognitive changes can be subtle and arduous to detect [1]. Along with biomarker positivity, a specific neuropsychological profile is of cardinal importance for diagnosing the disease and tracking its progression over time [2]. According to current criteria, individuals receive a diagnosis of dementia when the impairment affects at least two cognitive domains and interferes with daily living [3]. Conversely, a diagnosis of MCI is proposed when individuals manifest concerns about their changes in cognition (respect to a previous level of functioning), show mild deficits affecting one or more cognitive domains, but are otherwise independent on daily functional activities [4].

The use of validated neuropsychological measures, assessing episodic memory and other cognitive domains, is recommended for a diagnosis of MCI [4]. Notably, combining neuropsychological tests tapping different cognitive domains provides richer information compared to the use of a single test. For instance, the combined use of episodic memory measures with semantic fluency [5], or executive functions tests [6], or language, executive functions, and visuo-perceptual tests [7], achieves high sensitivity and specificity in predicting the conversion to AD. In this vein, several efforts have been dedicated to survey, select, and harmonize the available neuropsychological instruments to identify MCI subjects and AD patients. This goal has been pursued by several initiatives, for example in Europe [8], China [9], and U.S.A., in the latter case for both English and Spanish speaking individuals [10–13]. In particular, in the U.S.A., the Uniform Data Set initiative of the National Alzheimer's Coordinating Center [14] has led to the development and validation of a neuropsychological battery (UDSNB), now in its 3rd version [12]. In Italy, a recent initiative of the Virtual Dementia Institute of the Italian Network of Neuroscience and Neuro-Rehabilitation (RIN) has promoted the translation and adaptation of the UDSNB. The Italian version of the battery (I-UDSNB) is now available, together with normative data collected from 433 healthy individuals [15]. The battery is administrable via a tablet-based application and consists of tests covering different cognitive domains, i.e., memory, attention, language, executive functions, and visuospatial skills.

To foster the use of the I-UDSNB in clinical and research settings it is of pivotal importance to ascertain its clinical validity in staging the continuum between normal cognition, MCI, and AD. Previous studies have addressed the clinical validity of the UDSNB in MCI and AD cohorts, taking into account only some tests, such as the picture naming test [16, 17] or considering composite measures, such as an executive functioning score [18], and the global score [19].

The current multicenter Italian initiative was intended to explore the usefulness of the I-UDSNB to differentiate MCI and mild AD subjects from healthy individuals, as well as MCI from mild AD patients. To assess the diagnostic value of the I-UDSNB, we compared the raw test scores for the listed comparisons by means of a series of t-tests. Significant scores were entered in binary logistic regression models and in stepwise regressions to identify the test(s) most capable of differentiating between groups. For the scores emerging as significant in the previous analysis, we then calculated the Area Under the Curve (AUC), sensitivity, specificity, and cut-off values using Receiver Operating Characteristic (ROC) curve analyses.

The identification of the neuropsychological test(s) with the best diagnostic performance may offer noteworthy insights for the diagnosis, management, and monitoring of these patients in Memory Clinics.

## Methods

### Participants

A sample of 137 patients were recruited in thirteen Italian Hospitals belonging to the RIN network. Specifically, 65 subjects had received a diagnosis of MCI and 72 patients were diagnosed as mild probable AD. The diagnosis was formulated by expert clinicians in each center and was based on core clinical criteria [3, 4]. Data from the I-UDSNB were not used for diagnosis, and all the subjects were previously administered a comprehensive neuropsychological examination according to local practice.

Participants were excluded if they had prior/current cerebrovascular disorders; a history of traumatic brain injury, brain tumors, stroke; concomitant medical, sensory and/or motor deficits possibly affecting the performance; a history of alcohol and/or drug abuse; use of medications influencing cognitive functions. As we were specifically interested in the diagnostic usefulness of the test in the prodromal and early dementia stage, participants with a performance in the MMSE below 20 (score corrected for age and education) were excluded [20].

Biomarker information was available for 107 patients, specifically 47 with MCI and 60 with AD, and was collected by means of CSF in 67 cases (32 MCI, 35 AD), amyloid-PET in 36 cases (12 MCI, 24 AD), or both in 4 cases (3 MCI, 1 AD). In the case of PET data only a qualitative evaluation of amyloid positivity was available. For CSF data heterogeneity emerged in the measures considered and the reference values. Most of the centers assessed Aβ42, T-Tau, and P-Tau (in 8 centers each), Aβ42/Aβ40 was assessed in 5 centers, while other ratios between the aforementioned measures were collected in a minority of cases (i.e., 1 or 2 centers). Reference values were also variable. For Aβ42 normal reference values were > 600 pg/ml in 5 centers, > 640 pg/ml in 2 centers, while one center used both. For T-Tau normal reference values were < 404 pg/ml in 4 centers, < 580 pg/ml in 2 centers, < 450 pg/ml in 1 center, and > 275 pg/ml in 1 center. For P-Tau, reference values were < 56.50 pg/ml in most centers (*n* = 4), < 61 pg/ml in 2 centers, < 50 pg/ml and < 63 pg/ml in one center each. Finally, Aβ42/Aβ40 normal reference values were > 0.07 in all the centers. In total, 98.1% of patients with available biomarker information (i.e., 105 out of 107) showed positivity for AD, and specifically 95.7% (i.e., 45 out of 47) of MCI patients and 100% (i.e., 60 out of 60) of AD patients.

MCI and AD groups were matched for age (t(135) = 0.353, *p* = 0.725), years of education (t(135) = 0.366, *p* = 0.907), and sex (Chi-square = 0.118, *p* = 0.863). A lower score in the Mini-Mental State Examination (MMSE) was found in AD compared to MCI (t(135) = 7.795, *p* < 0.001). See Table 1 for the samples’ demographic information.

**Table 1:** Demographic information of the patients

	**AD (*n* = 72)**	**MCI (*n* = 65)**	**All sample (*n* = 137)**	**Difference between MCI and AD (*p*-value)**
age mean (SD)	74.04 (6.29)	74.40 (5.51)	74.21 (5.92)	0.725
years of education mean (SD)	10.49 (4.24)	11.12 (3.94)	10.79 (4.10)	0.907
MMSE mean (SD)	22.91 (2.79)	26.33 (2.29)	24.53 (3.07)	< 0.001
number of females/males	40/32	38/27	78/59	0.863

The number of participants, the mean and standard deviation of age, education (expressed in years), and MMSE, the number of females and males, and the *p*-values of the comparison between MCI and AD groups are reported; *AD *Alzheimer’s Disease, *MCI *Mild Cognitive Impairment, *SD *Standard deviation, *MMSE *Mini-Mental State Examination (corrected score, [21])

The study was approved by the local ethics committees in compliance with the provisions of the Declaration of Helsinki. All patients gave written informed consent to participate.

Data from 137 healthy controls (HC) from our previous norming study [15] were also included, divided into two groups (*n* = 65 and *n* = 72, respectively), and used for the comparison with MCI and AD groups (see below). The two groups of HC were matched between each other, and with the MCI and AD samples, respectively, for age, years of education, and sex (all p-values at least > 0.156).

### I-UDSNB administration

All the participants were administered the I-UDSNB, encompassing tests assessing memory, language, executive functions, processing speed, and visuo-constructional abilities. The battery includes the following tests (in order of administration): Craft Story, Benson Figure (Copy, Recall), Digit Span Forward and Backward, Semantic Fluency, Trail Making Test A and B (TMT-part A, TMT-part B), Picture Naming, Phonemic Fluency, and a short encoding controlled cued recall test (Five Words Test). The administration of the I-UDSNB takes approximately 45 min and it is supported by a newly developed tablet-based application, aiding the experimenter in administration and scoring [15].

### Data analysis

Data of TMT-part A and part B were excluded for 3 (1 AD and 2 MCI) and 10 patients (6 AD, 4 MCI), respectively, who were not able to perform the task, i.e. they were unable to follow task instructions.

The analyses proceeded according to three steps.

1. We assessed the differences between a) MCI and HC, b) AD and HC, and c) MCI and AD by comparing the raw scores in each test (*n* = 54 scores), using Mann–Whitney non parametric tests with Bonferroni correction (α = 0.05/54 = 0.0009). Non parametric tests were adopted because the majority of data were skewed and showed a non-normal distribution. A maximum of six significant scores in different tests with the highest effect size (Hedges' g) were selected for further analyses. We decided to keep six scores in order to approximately maintain a 1:10 ratio between the number of independent variables (i.e., the selected scores) and the number of observations per group in the regression models described below. The criterion of choosing scores belonging to different tests was intended to avoid multicollinearity.
2. The six significant scores were entered as predictors in a binary logistic regression, with the group membership as dependent variable, i.e., a) MCI and HC, b) AD and HC, and c) MCI and AD, respectively. Significant predictors were then used in a stepwise binary logistic regression. Specifically, they were entered following all the possible order combinations (e.g., first predictor A, then predictor B, and vice-versa), to ascertain whether each score would have significantly improved group membership prediction. Significant predictors were then retained for the third step.
3. Receiver Operating Characteristic (ROC) analyses were adopted to assess the ability of the scores selected from the previous analysis to differentiate between: a) MCI and HC, b) AD and HC, and c) MCI and AD, respectively. In order to facilitate the comparison between tests, whenever a higher score corresponded to a worse performance (e.g., TMT), this was inverted prior to the analysis. We evaluated the ability of the scores in performing the diagnostic classification using the Area Under the Curve (AUC). Significant AUC were used to estimate the optimal cut-off, calculated as the trade-off between sensitivity and specificity by means of the Youden Index (J) (i.e., sensitivity + specificity -1). We considered as acceptable the values of J above 0.5 [22], otherwise the cut-off was manually determined.

ROC analyses were also performed taking into account a composite measure obtained by summing the scores emerging as significant in point 1 described above. Whenever a higher score corresponded to a worse performance (e.g., TMT), this was inverted prior to entering the composite measure.

The same analyses were conducted taking into account only the sub-sample of patients showing biomarker positivity, i.e. 45 MCI and 60 AD patients. Biomarker positive patients and patients without biomarker information did not show any significant difference in demographic variables (age, years of education, and sex), in MMSE, and in the I-UDSNB tests scores; additionally, biomarker positive MCI and biomarker positive AD were matched between each other and with the respective control group for demographic variables (age, years of education, and sex).

## Results

### MCI vs HC

**Fig. 1:**
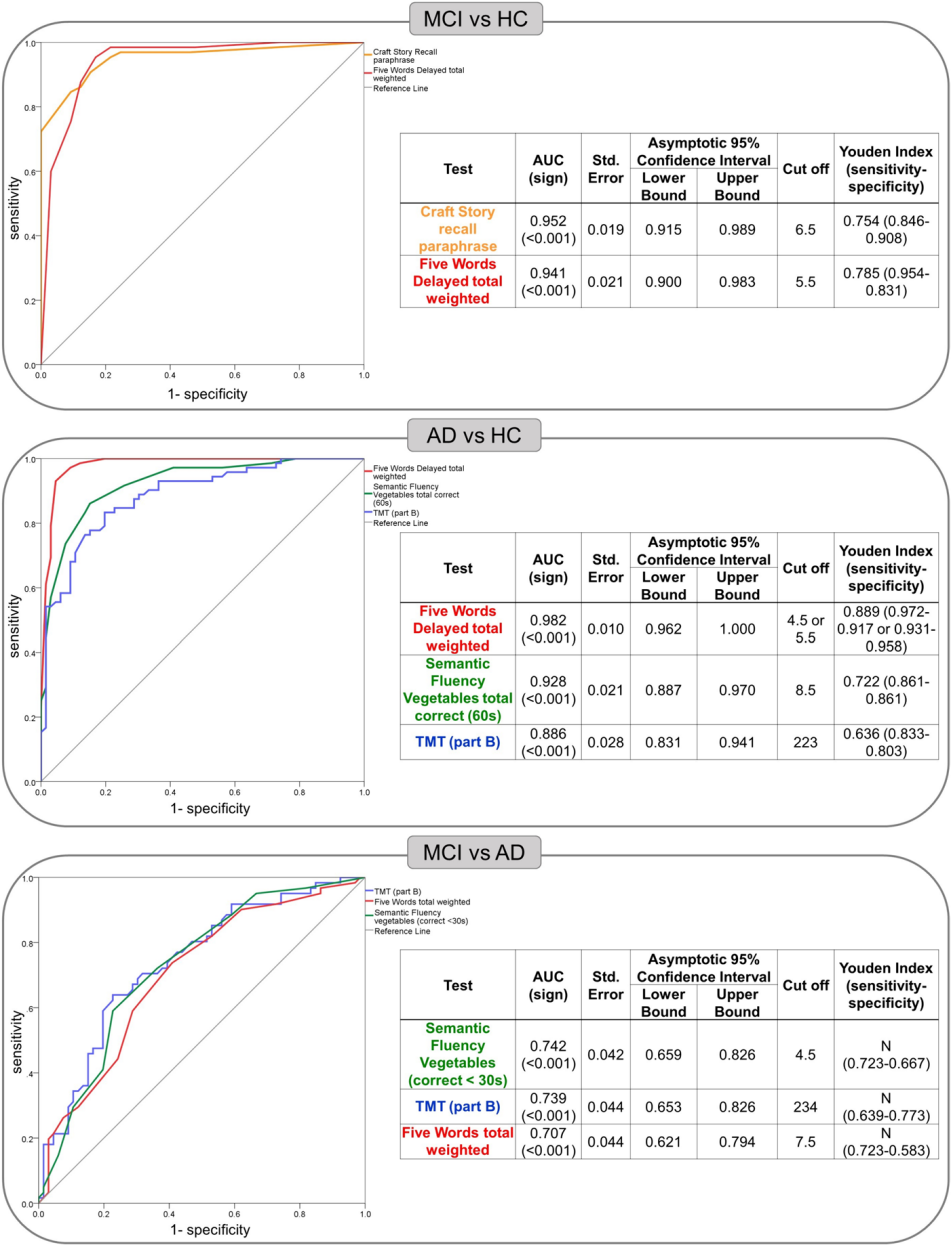
Results of the Receiver Operating Characteristic (ROC) analysis for the comparison between **a** MCI and HC, **b** AD and HC, and **c** MCI and AD; HC = healthy controls; MCI = Mild Cognitive Impairment; AD = Alzheimer’s Disease; AUC = area under the curve; N = Youden Index below the reference value of 0.5

The six significant scores with the highest effect size unveiling differences between MCI and HC were: Craft Story recall paraphrase (g = 2.632), Five Words test delayed total weighted recall (g = 2.593), Benson figure recall (g = 1.896), Semantic Fluency total correct score (g = 1.412), TMT (part B-A) (g = 1.121), and Picture Naming total correct score (g = 1.002). After being entered in the binary logistic regression, only Craft Story recall paraphrase (B = -0.293, *p*-value = 0.008) and Five Words test delayed total weighted recall (B = -0.447, *p*-value = 0.014) could significantly predict group membership (i.e., MCI, HC). In the stepwise binary logistic regression both of them significantly contributed to the prediction and were consequently retained for the ROC analysis. The latter unveiled that Craft Story recall paraphrase and Five Words test delayed total weighted recall differentiated MCI from HC with high sensitivity (0.846 and 0.954, respectively) and specificity (0.908 and 0.831, respectively). See Fig. 1 for the results of the ROC analysis.

The composite measure also significantly differentiated between MCI and HC (AUC: 0.879, *p*-value < 0.001), with a sensitivity of 0.862 and a specificity of 0.803.

The analyses including only biomarker positive MCI patients identified the same six scores emerging in the main analysis and significantly differentiating patients and HC as suggested by the non parametric tests. When considering the single scores for the subsequent analyses, there were no significant results, with only a trend towards significance in the binary logistic regression analysis for Craft Story recall paraphrase (*p*-value = 0.077) and Five Words test delayed total weighted recall (*p*-value = 0.080). Conversely, the composite measure successfully differentiated between biomarker positive MCI patients and HC (AUC: 0.901, *p*-value < 0.001), with a sensitivity of 0.862 and a specificity of 0.881.

### AD vs HC

The six significant scores with the highest effect size differentiating AD and HC were: Five Words test delayed total weighted recall (g = 3.788), Craft Story recall paraphrase (g = 2.661), Benson figure recall (g = 2.136), Semantic Fluency vegetables total correct score (g = 2.048), TMT (part B) (g = 1.623), and Picture Naming correct without cue score (g = 1.378). After being entered in the binary logistic regression, only Five Words test delayed total weighted recall (B = -1.126, *p*-value = 0.003), Semantic Fluency vegetables total correct score (B = -0.634, *p*-value = 0.019), and TMT (part B) (B = 0.020, *p*-value = 0.026), significantly predicted group membership (i.e., AD, HC). In the stepwise binary logistic regression all of them significantly contributed to the prediction and were used for the ROC analysis. The latter indicated that Five Words test delayed total weighted recall (sensitivity: 0.972 or 0.931, specificity: 0.917 or 0.958, see Fig. 1 for the respective cut-off value), Semantic Fluency vegetables total correct score (sensitivity: 0.861, specificity: 0.861), and TMT (part B) (sensitivity: 0.833, specificity: 0.803) successfully differentiated AD and HC. See Fig. 1 for the results of the ROC analysis.

The composite measure also significantly differentiated between AD and HC (AUC: 0.918, *p*-value < 0.001), with a sensitivity of 0.806 and a specificity of 0.894. The analyses taking into account only biomarker positive AD patients led to comparable results (all sensitivity and specificity values above 0.764).

### MCI vs AD

Five scores significantly differentiated MCI and AD showing the highest effect size: TMT (part B) (g = 0.887), Semantic Fluency vegetables (correct < 30 s) (g = 0.878), Five Words total weighted recall (g = 0.768), Phonemic Fluency letter F (correct < 30 s) (g = 0.587), and Picture Naming correct without cue score (g = 0.577). After being entered in the binary logistic regression, TMT (part B) (B = 0.006, *p*-value = 0.003), Five Words total weighted recall (B = -0.135, *p*-value = 0.038), and Semantic Fluency vegetables (correct < 30 s) (B = -0.192, *p*-value = 0.043) could significantly predict group membership (i.e., MCI, AD). In the stepwise binary logistic regression all of them significantly contributed to the prediction and were thus retained for the ROC analysis. The latter suggested that TMT (part B), Five Words total weighted recall, and Semantic Fluency vegetables (correct < 30 s) differentiated MCI from AD with good sensitivity (0.639, 0.723, and 0.723, respectively) and specificity (0.773, 0.583, and 0.667, respectively). See Fig. 1 for the results of the ROC analysis.

The composite measure also significantly differentiated between MCI and AD (AUC: 0.749, *p*-value < 0.001), with a sensitivity of 0.639 and a specificity of 0.803.

The analyses including only biomarker positive patients suggested that Semantic Fluency vegetables total correct score (g = 0.924) and TMT (part B) (g = 0.779) differentiated MCI and AD with the highest effect size, and the regression analyses suggested that both significantly contributed to the group membership prediction. ROC analysis indicated that Semantic Fluency vegetables total correct score and TMT (part B) differentiated MCI from AD with acceptable sensitivity (0.689 and 0.619, respectively) and specificity (0.773 and 0.617, respectively). The composite measure also significantly differentiated between biomarker positive MCI and AD (AUC: 0.725, *p*-value < 0.001), with a sensitivity of 0.619 and a specificity of 0.839.

See Supplementary Information for the descriptive statistics of the HC, MCI, and AD groups in the I-UDSNB tests, the percentage of patients showing a pathological performance according to the cut-off established from the normative data, and the results of the non-parametric tests comparing a) MCI and HC, b) AD and HC, and c) MCI and AD.

## Discussion

Our findings indicate high sensitivity and specificity of the I-UDS neuropsychological battery for the diagnosis of MCI and mild AD, and for differentiating between the two patients’ groups. The study sample had been diagnosed in 13 research hospitals of the Italian Ministry of Health Network of Neuroscience and Neuro-Rehabilitation across Italy, using the neuropsychological protocols currently adopted within each center. Our results suggest that the I-UDSNB is a suitable tool for the harmonized assessment of subjects with suspected cognitive impairment. Considering the referral diagnosis as the gold standard, all the probable AD patients had an impaired performance in at least one I-UDS subtest, and only 1 out of 65 MCI subjects had a profile fully within normal limits. As expected, the scores with the highest diagnostic value in the case of MCI individuals were those of verbal episodic memory tests. In the case of patients with a clinical diagnosis of AD dementia, tests evaluating semantic memory (semantic fluency) and executive functioning (TMT-part B), in association with a verbal episodic memory score, gave the highest contribution in differentiating AD from MCI and HC.

Our results fit well with the current literature, indicating that disturbances in episodic memory, namely in the ability to learn, store, and retrieve new information, represent the most common hallmark of MCI [23] and AD. Of note, verbal episodic memory performance is related to grey matter volume in medial temporal lobe regions, especially the entorhinal and hippocampal cortices [24, 25], known to show the earliest histological alterations during the course of AD [26, 27].

Despite the importance of episodic memory assessment, there is still a lack of consensus on which neuropsychological test is the most suitable instrument to diagnose MCI and AD. According to some proposals, the Free and Cued Selective Reminding Test (FCSRT) outperforms other verbal episodic memory tests, e.g., word list and paragraph recall, in differentiating MCI from AD [28], and in predicting amyloid positivity in MCI [29]. This superiority is advocated in virtue of two aspects characterizing the FCSRT, derived from the Grober-Buschke paradigm [30], namely the possibility to control for encoding and the presence of semantic cues during retrieval, thereby allowing to isolate and target the affected memory storage (versus retrieval) abilities. Amyloid status in MCI is indeed predicted by a diminished sensitivity to semantic cuing [31], which is progressively reduced as AD reaches the most advanced stages [32]. Nevertheless, the superiority of cued recall tests has been deemed as controversial [33] or nonexistent [34] by literature revisions. The latter work indeed concludes that all tests targeting verbal episodic memory, independently of material (e.g., word list vs paragraph) and test conditions (e.g., immediate vs delayed recall, presence vs absence of encoding and of cuing) show high sensitivity and specificity in predicting the progression from MCI to AD [34]. The discrepant results may be motivated by different factors, such as the heterogeneity of the patients’ groups, including disease severity as well as the qualitative profile of the impaired cognitive domains [33, 34]. In the current study, our results suggest that the Five Words test outperforms the logical memory test in the diagnostic classification of AD patients. Conversely, we found a comparable diagnostic performance of cued word recall (Five Words test) and of logical memory (Craft Story) in a population of MCI-core clinical criteria patients [4]. We suggest that the diagnostic superiority of cued word recall and its ability to isolate hippocampal damage are reduced in the case of a heterogeneous population of MCI patients defined on the basis of core clinical criteria (see, for example, [35]). Of note, when restricting the analyses to biomarker positive MCI individuals, even if a composite measure still differentiated between patients and controls, no significant results were found considering the single test scores, plausibly due to the reduced sample size as well as to the heterogeneity in the measures and the reference values used to assess biomarker positivity among centers.

Notwithstanding episodic memory deficits are usually the first and most prominent symptom, additional cognitive disturbances may be present from the beginning of the disease or may eventually appear and worsen during the progression of AD pathology to other brain structures [26]. This is clearly indicated by the high diagnostic value of semantic fluency and TMT-part B for the diagnosis of mild AD, and specifically to differentiate AD from both HC and MCI individuals.

For instance, impairments in semantic fluency have been reported in AD and usually ascribed to a deterioration of semantic knowledge [36, 37], as suggested by the associated hypometabolic pattern encompassing the temporal areas implicated in conceptual processing, e.g. the inferior temporal gyrus [38]. On the other hand, category fluency typically undergoes only a subtle decline in MCI [39, 40], and the underlying cause remains debated and attributed either to a deterioration of semantic knowledge [41], or to difficulties in lexical retrieval [42].

Additionally, in line with our results, deficits in TMT-part B have been previously reported in AD [43], fitting into the set of impairments that affect the multiple facets of executive functions, encompassing attention [44], working memory [45], inhibitory control, and cognitive flexibility [46]. These deficits have been suggested to correlate with the emergence of impairments in daily living [47], and to reflect tau deposition [48], as well as structural and functional alterations in fronto-parietal networks [44, 49]. In MCI, executive functions abilities may be compromised, albeit to a lesser extent than AD [44, 45], or preserved but associated with compensatory neural activations [50].

We acknowledge some limitations of this study. First, we included only mild AD patients and, although the I-UDSNB tests and the composite measures allowed to distinguish between the considered groups, the inclusion of AD patients with a more advance stage of dementia would have likely resulted in a better differentiation between MCI and AD. Moreover, heterogeneity emerged in the measures and reference values used to assess biomarker positivity across the centers, that may account for the absence of effects when restricting the analyses to biomarker positive MCI individuals, specifically when considering the individual tests scores. However, also in this case, a composite measure successfully differentiated between groups. An additional limitation pertains to the inclusion of a sample of healthy controls from which we previously obtained the normative data, which may represent a source of bias in the results and partially account for the overall high sensitivity and specificity in differentiating patients from HC. Finally, we have to mention that data on TMT-part A and B were excluded for 3 and 10 patients, respectively, thus overall reducing the sample size.

## Conclusion

In conclusion, the current multicenter study speaks in favor of the validity of the I-UDSNB as a harmonized and concise battery, able to adequately diagnose both MCI and AD patients. Limitations for MCI diagnosis [19], possibly due to the use of a logical memory test as a unique assessment of verbal episodic memory, were likely overcome by the addition of a brief cued word recall test, allowing to achieve high diagnostic sensitivity and specificity.

Finally, this study represents a direct continuation of our previous work leading to the creation of I-UDSNB and the collection of normative data [15]. Future initiatives may be dedicated to the employment of the I-UDSNB to address the longitudinal tracking of cognitive change, and to design modules for other types of dementia, e.g. Lewy bodies dementia and frontotemporal lobar degeneration.

### Supplementary Information

**Supplementary Material 1.**

## Data Availability

The datasets used and/or analyzed during the current study are available from the corresponding author after a reasonable request.

## Abbreviations

AD: Alzheimer’s Disease
MCI: Mild Cognitive Impairment
UDSNB: Neuropsychological Test Battery of the Uniform Data Set
I-UDSNB: Italian adaptation of Neuropsychological Test Battery of the Uniform Data Set
RIN: Italian Network of Neuroscience and Neuro-Rehabilitation
TMT: Trail Making Test
MMSE: Mini Mental State Examination
SD: Standard deviation
