# Supplementary material for: Clinical validity of the Italian adaptation of the Uniform Data Set Neuropsychological Test Battery (I-UDSNB) in Mild Cognitive Impairment and Alzheimer’s Disease
Source: Alzheimers Res Ther. 2024 May 4;16:98. doi: 10.1186/s13195-024-01465-0 (PMC11069160; doi:10.1186/s13195-024-01465-0)
Supplement: Supplementary file 1 — Supplementary Material 1. [file 13195_2024_1465_MOESM1_ESM.docx]

**Supplementary Information**

**Table 1.** Descriptive statistics (mean and standard deviation) of the raw scores of HC (respectively matched with MCI and matched with AD), MCI and AD patients in each test of the I-UDSNB, percentage of patients showing a pathological performance according to the cut-off established from the normative data, and results of the non-parametric tests comparing a) MCI and HC, b) AD and HC, c) MCI and AD; SD = standard deviation, AD = Alzheimer’s Disease, MCI = Mild Cognitive Impairment, % = percentage, - = no cut off, * = significant, Bonferroni-corrected.

|  | **HC matched with MCI** | | **HC matched with AD** | | **MCI** | | | **AD** | | | **HC vs MCI** | **HC vs AD** | **MCI vs AD** |
| --- | --- | --- | --- | --- | --- | --- | --- | --- | --- | --- | --- | --- | --- |
| **test** | **raw score mean** | **raw score SD** | **raw score mean** | **raw score SD** | **raw score mean** | **raw score SD** | **% pathological subjects** | **raw score mean** | **raw score SD** | **% pathological subjects** | **Mann-Whitney U (p-value)** | **Mann-Whitney U (p-value)** | **Mann-Whitney U (p-value)** |
| **Craft Story** |  |  |  |  |  |  |  |  |  |  |  |  |  |
| Immediate verbatim score | 13.22 | 6.02 | 12.51 | 5.58 | 7.45 | 4.76 | 26.15 | 5.24 | 3.35 | 29.17 | 963.5 (<0.0009)* | 688 (<0.0009)* | 1715 (0.007) |
| Immediate paraphrase score | 12.51 | 4.11 | 12.00 | 4.37 | 3.74 | 3.59 | 66.15 | 3.03 | 2.78 | 81.94 | 248.5 (<0.0009)* | 243 (<0.0009)* | 2186.5 (0.504) |
| Recall verbatim score | 10.11 | 4.94 | 9.83 | 5.51 | 3.20 | 4.08 | 44.62 | 1.88 | 3.37 | 66.67 | 598.5 (<0.0009)* | 486.5 (<0.0009)* | 1803 (0.013) |
| Recall paraphrase score | 11.20 | 4.49 | 10.86 | 4.48 | 1.66 | 2.48 | 78.46 | 1.28 | 2.43 | 90.28 | 204 (<0.0009)* | 164.5 (<0.0009)* | 2109 (0.265) |
| **Five Words Test** |  |  |  |  |  |  |  |  |  |  |  |  |  |
| Immediate free recall | 4.26 | 0.69 | 4.21 | 0.84 | 3.03 | 1.19 | 29.23 | 2.28 | 1.18 | 55.56 | 815.5 (<0.0009)* | 523 (<0.0009)* | 1475 (<0.0009)* |
| Immediate cued recall | 0.69 | 0.68 | 0.69 | 0.74 | 1.08 | 0.92 | 7.69 | 0.94 | 0.95 | 8.33 | 1641 (0.018) | 2260 (0.154) | 2130 (0.337) |
| Immediate total recall | 4.95 | 0.21 | 4.90 | 0.30 | 4.11 | 1.09 | 52.31 | 3.22 | 1.37 | 79.17 | 1112 (<0.0009)* | 659 (<0.0009)* | 1451 (<0.0009)* |
| Immediate total-weighted recall | 9.22 | 0.76 | 9.11 | 1.01 | 7.14 | 2.08 | 43.08 | 5.50 | 2.37 | 68.06 | 691 (<0.0009)* | 391.5 (<0.0009)* | 1368 (<0.0009)* |
| Delayed free recall | 3.75 | 1.16 | 3.58 | 1.20 | 0.69 | 1.22 | 78.46 | 0.22 | 0.75 | 91.67 | 263.5 (<0.0009)* | 138.5 (<0.0009)* | 1850.5 (0.002) |
| Delayed cued recall | 0.91 | 0.95 | 1.04 | 0.93 | 1.17 | 1.13 | 13.85 | 0.89 | 1.21 | 13.89 | 1856 (0.207) | 2155 (0.064) | 1914 (0.052) |
| Delayed total recall | 4.66 | 0.71 | 4.63 | 0.66 | 1.86 | 1.69 | 67.69 | 1.11 | 1.42 | 84.72 | 362.5 (<0.0009)* | 193 (<0.0009)* | 1699.5 (0.004) |
| Delayed total-weighted recall | 8.42 | 1.68 | 8.21 | 1.69 | 2.55 | 2.72 | 83.08 | 1.33 | 1.93 | 95.83 | 247.5 (<0.0009)* | 93 (<0.0009)* | 1671.5 (0.003) |
| Total free recall | 8.02 | 1.56 | 7.79 | 1.74 | 3.72 | 1.86 | 73.85 | 2.50 | 1.57 | 90.28 | 216.5 (<0.0009)* | 138.5 (<0.0009)* | 1348 (<0.0009)* |
| Total cued recall | 1.60 | 1.26 | 1.74 | 1.24 | 2.25 | 1.71 | 12.31 | 1.83 | 1.91 | 15.28 | 1684 (0.041) | 2420.5 (0.482) | 1930 (0.072) |
| Total recall | 9.62 | 0.86 | 9.53 | 0.80 | 5.97 | 2.36 | 67.69 | 4.33 | 2.39 | 86.11 | 341 (<0.0009)* | 147 (<0.0009)* | 1474.5 (<0.0009)* |
| Total-weighted recall | 17.63 | 2.18 | 17.32 | 2.40 | 9.69 | 3.88 | 78.46 | 6.83 | 3.57 | 94.44 | 205 (<0.0009)* | 92.5 (<0.0009)* | 1370 (<0.0009)* |
| **Picture Naming** |  |  |  |  |  |  |  |  |  |  |  |  |  |
| Correct without cue score | 30.28 | 2.29 | 29.88 | 3.07 | 25.75 | 6.02 | 40 | 21.71 | 7.80 | 66.67 | 997.5 (<0.0009)* | 715.5 (<0.0009)* | 1556 (<0.0009)* |
| Correct with cue score | 0.35 | 0.55 | 0.26 | 0.55 | 0.83 | 1.10 | 26.15 | 1.19 | 1.33 | 34.72 | 1470.5 (0.001) | 1149.5 (<0.0009)* | 1888 (0.046) |
| Correct total score | 30.62 | 2.08 | 30.14 | 2.77 | 26.58 | 5.30 | 41.54 | 22.90 | 7.58 | 63.89 | 997 (<0.0009)* | 754.5 (<0.0009)* | 1602.5 (0.001) |
| **Semantic Fluency** |  |  |  |  |  |  |  |  |  |  |  |  |  |
| Animals correct score (< 30 s) | 12.18 | 3.74 | 12.40 | 3.97 | 8.97 | 4.15 | 20 | 6.68 | 3.24 | 34.72 | 1125 (<0.0009)* | 667.5 (<0.0009)* | 1596.5 (0.001) |
| Animals correct score (> 30 s) | 6.77 | 3.87 | 5.86 | 3.42 | 3.29 | 2.18 | 12.31 | 2.75 | 2.48 | 23.61 | 865.5 (<0.0009)* | 1176.5 (<0.0009)* | 1940 (0.082) |
| Animals total correct score (60 s) | 18.95 | 6.45 | 18.26 | 5.86 | 12.26 | 4.50 | 18.46 | 9.43 | 4.89 | 36.11 | 784.5 (<0.0009)* | 569.5 (<0.0009)* | 1492.5 (<0.0009)* |
| Animals perseverations | 0.65 | 1.19 | 0.71 | 1.20 | 1.11 | 1.48 | 12.31 | 0.99 | 1.31 | 8.33 | 1720 (0.045) | 2187 (0.076) | 2275 (0.765) |
| Animals violations | 0.22 | 0.45 | 0.47 | 1.27 | 0.15 | 0.48 | 0 | 0.13 | 0.47 | 1.39 | 1933.5 (0.183) | 2026.5 (0.001) | 2282.5 (0.626) |
| Vegetables correct score (< 30 s) | 8.12 | 2.86 | 9.15 | 3.29 | 6.09 | 2.63 | 10.77 | 3.75 | 2.70 | 45.83 | 1240.5 (<0.0009)* | 522.5 (<0.0009)* | 1205.5 (<0.0009)* |
| Vegetables correct score (> 30 s) | 3.52 | 2.33 | 3.58 | 2.32 | 1.58 | 1.33 | 27.69 | 1.32 | 1.24 | 30.56 | 1005.5 (<0.0009)* | 1028.5 (<0.0009)* | 2075 (0.239) |
| Vegetables total correct score (60s) | 11.65 | 3.85 | 12.74 | 4.14 | 7.68 | 3.05 | 12.31 | 5.07 | 3.30 | 48.61 | 876 (<0.0009)* | 373 (<0.0009)* | 1269 (<0.0009)* |
| Vegetables perseverations | 0.54 | 0.89 | 0.57 | 0.87 | 0.66 | 1.05 | 7.69 | 0.57 | 0.87 | 4.17 | 2001.5 (0.549) | 2579 (0.952) | 2263 (0.701) |
| Vegetables violations | 0.77 | 1.30 | 0.61 | 1.11 | 0.63 | 1.01 | 0 | 0.46 | 1.01 | 4.17 | 2050 (0.737) | 2423.5 (0.400) | 2082 (0.174) |
| Total correct score (60 s) | 30.60 | 8.34 | 31.00 | 8.87 | 19.94 | 6.67 | 29.23 | 14.50 | 7.50 | 65.28 | 610.5 (<0.0009)* | 379 (<0.0009)* | 1320 (<0.0009)* |
| Total perseverations | 1.18 | 1.42 | 1.28 | 1.65 | 1.77 | 2.13 | 10.77 | 1.56 | 1.85 | 8.33 | 1888 (0.277) | 2318 (0.254) | 2290 (0.824) |
| Total violations | 0.98 | 1.42 | 1.08 | 1.96 | 0.78 | 1.14 | 0 | 0.58 | 1.16 | 0 | 1953 (0.416) | 2138 (0.038) | 2037.5 (0.129) |
| **Phonemic Fluency** |  |  |  |  |  |  |  |  |  |  |  |  |  |
| Letter F correct score (< 30 s) | 7.69 | 2.98 | 7.70 | 3.42 | 6.83 | 3.19 | 15.38 | 4.96 | 3.19 | 31.94 | 1796 (0.138) | 1402 (<0.0009)* | 1544 (<0.0009)* |
| Letter F correct score (> 30 s) | 4.68 | 2.23 | 4.10 | 2.77 | 3.80 | 2.53 | 9.23 | 3.24 | 2.29 | 15.28 | 1632.5 (0.024) | 2126.5 (0.081) | 2075.5 (0.250) |
| Letter F total correct score (60 s) | 12.37 | 4.51 | 11.86 | 5.33 | 10.63 | 4.52 | 15.38 | 8.19 | 4.66 | 41.67 | 1675.5 (0.041) | 1565 (<0.0009)* | 1586.5 (0.001) |
| Letter F perseverations | 0.38 | 0.65 | 0.33 | 0.58 | 0.88 | 1.18 | 18.46 | 0.72 | 1.14 | 15.28 | 1590.5 (0.006) | 2192 (0.056) | 2086 (0.227) |
| Letter F violations | 0.28 | 0.65 | 0.31 | 0.82 | 0.28 | 0.57 | 0 | 0.35 | 0.61 | 0 | 2079 (0.825) | 2362.5 (0.211) | 2199 (0.420) |
| Letter L correct score (< 30 s) | 6.88 | 2.73 | 6.17 | 3.23 | 5.38 | 2.47 | 16.92 | 4.03 | 2.52 | 31.94 | 1456 (0.002) | 1593 (<0.0009)* | 1648.5 (0.003) |
| Letter L correct score (> 30 s) | 3.88 | 2.27 | 2.97 | 2.33 | 2.74 | 1.80 | 15.38 | 2.14 | 1.78 | 20.83 | 1526 (0.006) | 2072.5 (0.048) | 1864.5 (0.038) |
| Letter L total correct score (60 s) | 10.75 | 4.33 | 9.15 | 4.87 | 8.12 | 3.35 | 13.85 | 6.17 | 3.71 | 25 | 1433.5 (0.001) | 1678 (<0.0009)* | 1563.5 (<0.0009)* |
| Letter L perseverations | 0.35 | 0.60 | 0.40 | 0.83 | 0.51 | 0.94 | 10.77 | 0.43 | 0.71 | 9.72 | 2043.5 (0.690) | 2455.5 (0.494) | 2337.5 (0.989) |
| Letter L violations | 0.37 | 0.76 | 0.53 | 0.92 | 0.58 | 1.13 | 7.69 | 0.81 | 1.38 | 9.72 | 1922 (0.259) | 2307.5 (0.190) | 2074 (0.186) |
| Total correct score (60 s) | 23.12 | 8.15 | 21.01 | 9.59 | 18.75 | 7.09 | 16.92 | 14.36 | 7.91 | 34.72 | 1520 (0.006) | 1544 (<0.0009)* | 1501 (<0.0009)* |
| Total perseverations | 0.74 | 0.92 | 0.74 | 1.11 | 1.38 | 1.74 | 13.85 | 1.15 | 1.39 | 8.33 | 1745.5 (0.067) | 2125.5 (0.044) | 2257 (0.707) |
| Total violations | 0.65 | 1.10 | 0.83 | 1.52 | 0.86 | 1.42 | - | 1.15 | 1.59 | - | 1953.5 (0.399) | 2218.5 (0.102) | 2060 (0.190) |
| **Benson Figure** |  |  |  |  |  |  |  |  |  |  |  |  |  |
| Copy | 14.58 | 2.04 | 15.08 | 1.84 | 14.02 | 3.15 | 23.08 | 11.56 | 5.33 | 37.5 | 2046.5 (0.753) | 1545 (<0.0009)* | 1746.5 (0.009) |
| Recall | 10.28 | 3.21 | 10.13 | 3.67 | 4.11 | 3.30 | 53.85 | 2.67 | 3.31 | 72.22 | 418.5 (<0.0009)* | 419.5 (<0.0009)* | 1669 (0.003) |
| **Digit Span Forward** |  |  |  |  |  |  |  |  |  |  |  |  |  |
| Number of correct trials | 5.95 | 1.62 | 5.86 | 1.74 | 5.55 | 2.00 | 7.69 | 5.18 | 1.79 | 5.56 | 1785.5 (0.122) | 2034.5 (0.024) | 2091.5 (0.277) |
| Span length | 5.52 | 0.92 | 5.50 | 0.99 | 5.31 | 1.14 | 4.62 | 5.13 | 1.01 | 1.39 | 1862 (0.220) | 2086 (0.034) | 2124.5 (0.333) |
| **Digit Span Backward** |  |  |  |  |  |  |  |  |  |  |  |  |  |
| Number of correct trials | 5.14 | 1.33 | 5.03 | 1.76 | 4.35 | 1.61 | 7.69 | 3.69 | 1.43 | 13.89 | 1478.5 (0.003) | 1504 (<0.0009)* | 1812.5 (0.020) |
| Span length | 3.94 | 0.79 | 3.97 | 1.06 | 3.54 | 0.95 | 6.15 | 3.21 | 0.89 | 15.28 | 1628 (0.016) | 1576.5 (<0.0009)* | 1869 (0.029) |
| **Trail Making Test** |  |  |  |  |  |  |  |  |  |  |  |  |  |
| Part A (seconds) | 54.89 | 28.86 | 58.38 | 26.77 | 70.71 | 33.46 | 12.7 | 104.38 | 65.60 | 29.58 | 1264.5 (<0.0009)* | 1159 (<0.0009)* | 1461 (<0.0009)* |
| Part B (seconds) | 139.65 | 61.83 | 156.75 | 80.33 | 229.05 | 101.23 | 36.07 | 334.02 | 131.94 | 65.15 | 819.5 (<0.0009)* | 541.5 (<0.0009)* | 1049 (<0.0009)* |
| Part B-A (seconds) | 84.75 | 43.31 | 98.38 | 62.62 | 159.79 | 84.21 | 50.82 | 233.98 | 115.19 | 68.18 | 819 (<0.0009)* | 618 (<0.0009)* | 1200.5 (<0.0009)* |
